# Correlation of patient reported outcomes and physical exam screening for pelvic floor dysfunction: An opportunity for improved quality of life during chemoradiotherapy for cervical cancer

**DOI:** 10.1016/j.gore.2026.102083

**Published:** 2026-04-19

**Authors:** Sarah N. Badwan, Tyler R. McKinnish, L.Stewart Massad, Stephanie Markovina, Jessika Contreras, Julie Schwarz, Karry Brim, Brooke E. Sanders, Lindsay M. Kuroki, Andrea R. Hagemann, Carolyn K. McCourt, Premal H. Thaker

**Affiliations:** a Division of Gynecologic Oncology, Department of Obstetrics and Gynecology, Washington University School of Medicine, MO 63110, United States; b Department of Radiology, Washington University School of Medicine, MO 63110, United States

**Keywords:** Pelvic floor dysfunction, Pelvic floor, Triggerpoints

## Abstract

**Objective:**

To define the prevalence and time course of pelvic floor dysfunction (PFD) among cervical cancer patients receiving chemoradiotherapy (CRT).

**Methods:**

In a prospective study, women receiving curative-intent CRT were assessed for PFD at baseline, mid-treatment (3 weeks), completion (6 weeks), and follow-up (6 months) through pelvic exams and patient-reported outcome measures (PROMs). A standardized pelvic floor exam was performed at each time point by a trained oncologist, with pelvic floor pain defined as levator/obturator score > 3. Correlations between exam findings and PROM scores were analyzed using Spearman’s coefficient, and changes over time were assessed with ANOVA.

**Results:**

Twenty patients were enrolled, most with FIGO stage III disease, and received concurrent cisplatin. At baseline, 50% had PFD, improving to 25% by follow-up. EORTC QLQ-CX24 scores showed consistent positive correlations with exam findings at baseline (ρ = 0.465, p < 0.001), mid-treatment (ρ = 0.444, p < 0.001), and follow-up (ρ = 0.398, p < 0.001). PFIQ-7 demonstrated significant negative correlations, while PFDI-20 showed no consistent correlations. PROM trends indicated substantial improvement in EORTC QLQ-CX24 scores from pre-treatment to follow-up, paralleling exam findings.

**Conclusions:**

Half of patients with advanced cervical cancer exhibit PFD prior to CRT. While the lack of validated PROM thresholds limits their use for initial diagnosis, the EORTC QLQ-CX24 correlated most consistently with exams, highlighting its potential superiority as a non-invasive monitoring tool for PFD. Additional studies are warranted to determine if early intervention would benefit patients with pre-existing PFD and identify strategies for management of persistent dysfunction.

## Highlights

• Pelvic floor myofascial disorders are common among cervical cancer patients treated with radiation.
• Most cervical cancer patients with pelvic floor myofascial disorders improve acorss time with treatment.
• The EORTC QLQ-CX24 is well correlated with digital scoring in assessing severity of pelvic floor disorders.

## 1 Introduction

Pelvic floor dysfunction encompasses a broad constellation of symptoms and anatomic changes resulting in pain and dysfunction of the pelvic floor musculature (Grimes et al., 2023). These abnormalities may manifest as increased muscular activity, decreased activity, or poor coordination, leading to pelvic organ prolapse and a variety of urologic, gynecologic, and colorectal symptoms. Functionally, pelvic floor dysfunction can affect the anterior, middle, and posterior compartments of the pelvic floor, impairing quality of life.

Despite its prevalence and clinical significance, pelvic floor dysfunction remains underdiagnosed and insufficiently characterized in patients treated for gynecologic cancers. Most existing studies rely on retrospective surveys and lack objective baseline assessments or standardized physical examinations. While advanced diagnostic methods such as manometry and electromyography have been explored, they are not widely applied in clinical practice and lack validation against long-term functional outcomes (Braekken et al., 2021 Dec).

Women undergoing treatment for gynecologic malignancies represent a particularly vulnerable population. The cause of pelvic floor dysfunction in this group is likely multifactorial, and may include invasion or compression by malignancy, menopause, psychological stress, invasive procedures, and anticancer therapy including radiation therapy (RT) and chemotherapy (Cui et al., 2023 Jun). Pelvic RT, commonly administered as part of standard care for cervical and uterine cancers, may contribute to long-term pelvic floor injury, yet its specific impact on function remains unclear. In particular, survivors who received pelvic radiotherapy frequently report persistent urinary, bowel, and sexual dysfunction, as well as chronic pelvic pain, yet the pelvic floor has largely been ignored as a contributor to these symptoms, highlighting the need for targeted assessment and intervention (Cui et al., 2023 Jun). Advances in radiation delivery, including intensity modulated radiation therapy (IMRT) and split-field techniques, have reduced off-target exposure to non-target tissues in other malignancies; however, the impact of these approaches on the pelvic floor dose and function is unknown (Cai et al., 2023 Sep).

The absence of standardized, prospective evaluation protocols and comprehensive prevalence data limits understanding of pelvic floor dysfunction development, progression, and correlation with patient-reported outcomes in gynecologic cancer survivors. As survivorship increases, early detection and intervention to preserve pelvic floor function and minimize symptoms become increasingly important. This study addresses these gaps by prospectively evaluating pelvic floor dysfunction in women undergoing chemoradiotherapy for cervical cancer, with the aims of (1) determining the prevalence of pelvic floor dysfunction, (2) characterizing changes in pelvic floor function and symptoms over time, (3) assessing correlations between physical exam findings and patient-reported outcomes, and (4) evaluating the feasibility of standardized pelvic floor dysfunction assessments during active treatment.

## 2 Methods

This study was conducted at a university-affiliated radiation oncology and gynecology oncology clinics. The study was approved by the institutional review board, and all participants provided written informed consent prior to enrollment. Recruitment occurred from June 2023 to March 2025, until twenty participants had enrolled in the study. Eligible participants were English-speaking women aged ≥ 18 years with newly diagnosed locally advanced cervical cancer scheduled to receive curative-intent platinum-based chemoradiotherapy. Exclusion criteria included prior pelvic radiation, chronic non-cancer pain, inflammatory bowel disease, interstitial cystitis, or planned anti-estrogenic therapy. Enrollment reflects a convenience sample accrued before personnel changes required study closure.

Participants were identified prior to initial consultation with radiation oncology and consented at an in-person visit after discussion with the treating radiation oncologist. Participants then underwent standardized, prospective assessment at four time points: Baseline (prior to initiating treatment), midpoint (halfway through their treatment regimen), post-treatment (6 weeks following their last treatment), and at follow-up (6 months following their last treatment).

A single-digit, standard of care, physical examination with high interrater reliability was used to identify pelvic floor myofascial pain to determine pelvic floor dysfunction (Meister et al., 2019). Participants rated palpated muscle tenderness intensity on a 0–10 scale at five paired pelvic muscle locations. The sum of scores across locations yielded a composite pelvic floor dysfunction score, with pelvic floor dysfunction defined as a bilateral levator/obturator score of greater than 3, Examinations were performed by the treating physician (primarily radiation oncologists), in person, prior to the start of each treatment session, if applicable. Radiation oncology providers underwent a two-hour training session with a standardized patient led by a urogynecologist (CG) to ensure standardization of exam performance (Meister et al., 2019).

Concurrently, three validated PROMs were completed by participants: the European Organization for Research and Treatment of Cancer Quality of Life questionnaire cervical cancer (EORTC QLQ-CX24), Pelvic Floor Impact Questionnaire 7 (PFIQ 7), and Pelvic Floor Disability Index 20 (PFDI 20) (Barber et al., 2005 Jul, Greimel et al., 2006 Oct 15) [S1]. Questionnaires were administered on paper in person or by phone under standardized instructions. If questionnaires were unable to be completed at the same time as pelvic exam, a follow-up phone call within one week of the time of their scheduled pelvic exam and questionnaire was completed over the phone and responses were recorded on paper. Scoring followed each instrument’s published guidelines, where higher EORTC QLQ-CX24 scores indicated worse symptom burden and higher PFIQ-7/PFDI-20 scores reflected greater functional impact (Sun et al., 2015, Peerenboom et al., 2024 Jan). Demographic and clinical data including age, parity, menopausal status, comorbidity indices, and treatment parameters were collected from the electronic medical record.

Patients underwent staging with 18F-FDG-PET/CT and pelvic MRI when not contraindicated. Our institutional standard of care for definitive RT has been described previously and includes EBRT using split-field IMRT technique to treat the draining lymphatics to 5040 cGy in 180 cGy daily fractions, while the central pelvis receives 20 Gy with external beam radiation (71 cGy per day to the center of the pelvis). High dose rate brachytherapy boost was delivered with 3D-image guidance (MRI when not contraindicated) and intracavitary or hybrid interstitial-intracavitary tandem and ovoid implant to a nominal dose of 4380 cGy in 6 weekly fractions of 730 cGy, interdigitated with EBRT and beginning as early as week one of treatment (Kidd et al., 2010 Jul 15). This approach allows high-dose tumor coverage while minimizing radiation exposure to central pelvic structures including bladder and rectum (Sun et al., 2015, Peerenboom et al., 2024 Jan). Patients with pelvic sidewall invasion on imaging or otherwise unfavorable tumor geometry were treated with whole pelvic IMRT using a less-than whole uterus approach as previously described (Dyk et al., 2014). Brachytherapy boost dose in this case is reduced to 3000 cGy in 5 weekly fractions as stated above.

The primary outcome was the presence of pelvic floor dysfunction, as determined by pelvic exam. Secondary outcomes included PROM scores from the EORTC QLQ-CX24, PFIQ-7, and PFDI-20, and changes in these measures over time. Exposure variables were administration of chemoradiotherapy, radiation technique (whole pelvis vs. split-field IMRT), and time since treatment initiation. Potential confounders included age, parity, menopausal status, medical history, and surgical history.

Descriptive statistics were used to summarize patient demographics, clinical characteristics, and treatment parameters. Spearman’s rank correlation coefficient (ρ) was used to assess the strength and direction of associations due to non-normal distributions of PROM scores and exam scores. Changes in PROM and exam scores over time were analyzed using repeated-measures analysis of variance (ANOVA) to account for within-subject correlation across time points. Given the limited sample size (n = 20), formal multivariable adjustment was not performed. Potential confounders were summarized descriptively to allow assessment of potential influence on study outcomes. Exploratory subgroup analyses were planned for age (<50 vs ≥ 50 years), menopausal status, and disease stage; these are descriptive only due to small sample sizes.

## 3 Results

From June 2023 to March 2025, thirty-two participants were identified as potentially eligible, of whom 22 met inclusion criteria. Ten participants were excluded prior to enrollment: nine declined participation, were not interested, or did not respond to contact attempts, and one was excluded due to a history of irritable bowel syndrome. 20 eligible participants were enrolled and completed baseline assessments; Pelvic floor examinations were successfully completed in 85% (17/20) of planned assessments at treatment completion and 60% (12/20) at six-month follow-up, while PROM completion remained high throughout the study, with 90% (18/20) of participants completing surveys at the final time point., demonstrating the feasibility of standard assessments for pelvic floor dysfunction. Two initially enrolled participants were withdrawn from the study after their treatments were unexpectedly discontinued following baseline evaluation; two additional participants were enrolled to maintain the target enrollment of 20 participants. Of the 20 enrolled participants, all completed baseline physical examinations. Sixteen participants completed mid-treatment assessments. Nineteen participants completed post-treatment (6-week) assessments, and seventeen completed the 6-month follow-up. Twenty out of twenty patients completed PROMs at baseline, mid-treatment, and post-treatment, with eighteen out of twenty patients having completed the PROMs at the follow-up timepoint.

The average age was 52 years. Most participants were white (n = 16) and had squamous cell carcinoma (n = 18), (Table 1). The majority had squamous cell carcinoma, and a smaller proportion had adenocarcinoma. Disease stages ranged from FIGO (2019) stages I to IV, with most participants presenting with advanced stage III disease. Eighteen of twenty participants underwent split-field pelvic IMRT, and all received concurrent chemotherapy. Participants were queried about sexual trauma; none reported it. No adverse events were attributed to the study. Expected toxicities were consistent with standard chemoradiotherapy regimens.

**Table 1:** Demographics.

**Demographics**	
**Characteristic**	**n (%) or Mean ± SD**
**Age (years)**	52.4 ± 11.3
< 40	2 (10%)
40–49	7 (35%)
50–59	5 (25%)
60–69	6 (30%)
**Race**	
Black or African American	4 (20%)
White	16 (80%)
**Histology**	
Squamous Cell Carcinoma	18 (90%)
Adenocarcinoma	2 (10%)
**FIGO Stage**	
I	4 (20%)
II	3 (15%)
III	8 (40%)
IV	5 (25%)
**Menopausal Status**	
Pre-menopause	8 (40%)
Post-menopause (>1 yr)	12 (60%)
**Chemotherapy**	
Cisplatin	19 (95%)
Carboplatin	1 (5%)

Pelvic floor dysfunction was confirmed at baseline, in 10/20 participants (50%) 8/17 participants (47%) at completion of treatment, and 5/12 participants (41.6%) at 6-month follow-up [S2]. Of the five patients with pelvic floor dysfunction at 6-month follow-up, three patients did not have pelvic floor dysfunction at the time of their baseline examine, suggesting that they developed pelvic floor dysfunction while on therapy. Of the five participants with pelvic floor dysfunction at the time of their 6-month follow-up, three had pain scores > 9, suggesting severe pelvic floor dysfunction.

Exploratory subgroup analyses stratified by age, stage, and menopausal status were limited due to sample size. Repeated measures ANOVA showed a statistically significant effect of timepoint on scores with an effect size of 0.12 and p-value of 0.026, 15% of exams were not completed at completion of treatment and 40% of exams were not completed at 6-month follow-up [S2]. Missing exam assessments were primarily due to unscheduled or forgotten exams, patient declined for personal or comfort reasons, or lack of in-person follow-up with the treating provider. In order to not distress patients and respect their autonomy, they were not asked follow-up questions after their initial response as to whether they consent to the scheduled exam or not. While these patients could have been approached at a different time, the time points of the exams were determined based on standard of care follow-ups and procedures. Follow-up exams, while technically not impossible, may have been difficult to complete through participant approval to return to clinic to a non-standard of care or regularly scheduled appointment.

**Fig. 1:**
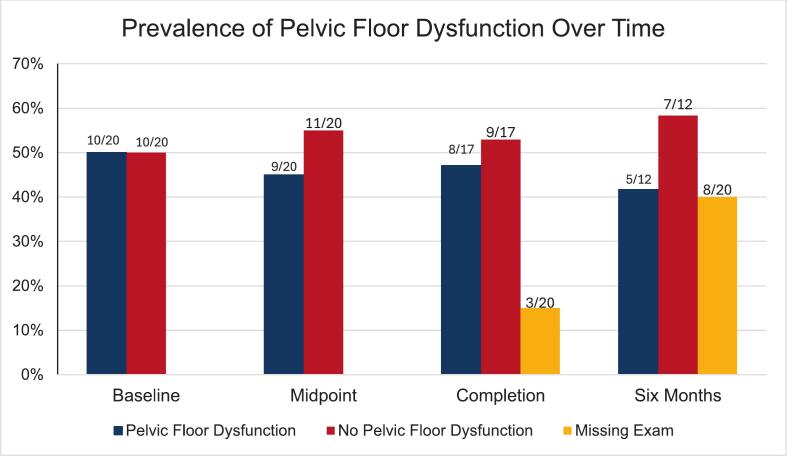
Prevalence in Pelvic Floor Dysfunction Over Time.

**Fig. 2:**
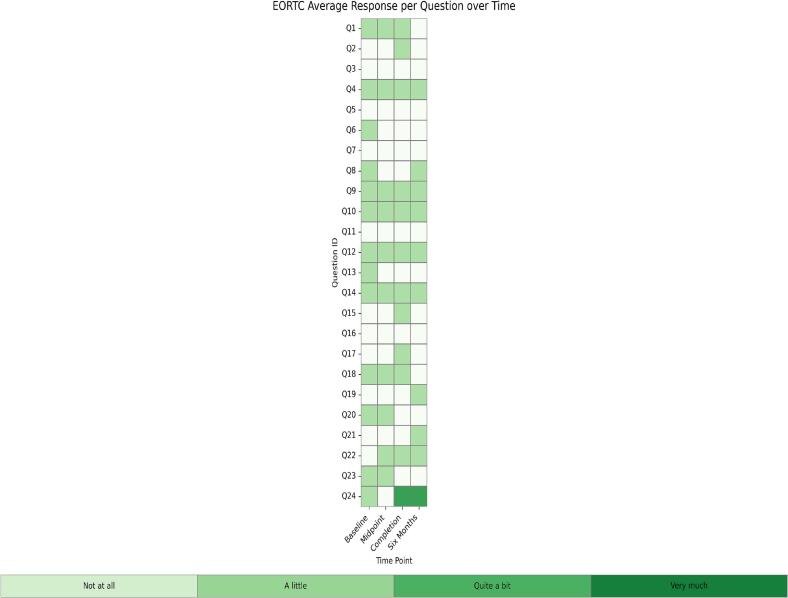

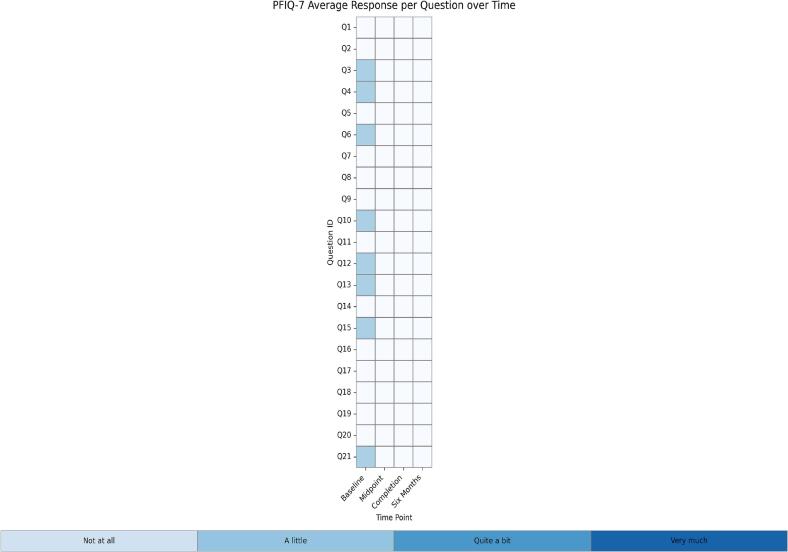

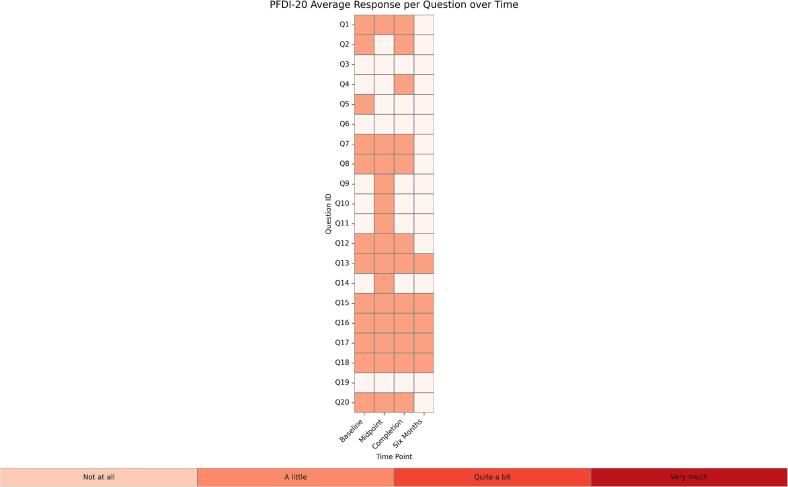
Heat Maps of Average QOL Response to Questions about Symptoms Among Individual Participants.

Median EORTC QLQ-CX24 scores improved from 45 at baseline to 25 at six months, reflecting reductions in general cramping, back pain, as well as vaginal bleeding, soreness, and discharge. PFIQ-7 scores showed modest improvement over the same period, capturing changes in the general physical and emotional impact of the diagnosis on daily life. While PFDI-20 scores did not show consistent trends across all items, some areas, particularly pelvic pressure, pain, and aspects of bowel function, demonstrated general improvement. Correlations between physical exam findings and PROMs were strongest for the EORTC QLQ-CX24 across all timepoints, with moderate positive correlations indicating agreement between subjective and objective measures of pelvic floor function. Pelvic floor dysfunction prevalence at each study timepoint demonstrates a gradual reduction in symptom burden over six months (Fig. 1). Average responses for each PROM item at each timepoint highlight areas of persistent symptoms and improvement across the study period (Fig. 2). Completion rates were highest at baseline at 100% and remained acceptable at subsequent time points, with 90% of patients having completed surveys at the six-month follow-up.

Post-treatment FDG-PET imaging showed partial or complete metabolic response to chemoradiotherapy treatment in nearly all participants. Only one patient did not respond favorably to treatment due to disease progression.

## 4 Discussion

In this prospective study, pelvic floor dysfunction was common at baseline in women undergoing chemoradiotherapy for cervical cancer, affecting half of all participants. The prevalence of dysfunction declined steadily during and after treatment, coinciding with clinical and imaging evidence of tumor response. These findings suggest that symptoms commonly attributed to pelvic floor dysfunction are not static and may, in part, reflect cancer-related pelvic pain that improves as the malignancy regresses. However, 25% of patients in this study had persistence of pain on exam six months after completion of treatment, at which time EORTC scores also remained elevated. This may indicate that pelvic floor dysfunction in this population do not always resolve with treatment and require rehabilitation; rehabilitation exercises reduce pelvic floor pain among previously radiated gynecologic cancer survivors with urinary incontinence (de Bem et al., 2026) but further study is needed to determine whether these results are generalizable to patients with newly diagnosed cervical cancer (de Bem et al., 2026). Standardized pelvic floor dysfunction assessments appeared feasible, as indicated by high exam completion rates, patient willingness, safety, and ease of integration into routine oncology visits. In contrast, other assessment strategies, while potentially more informative, often require specialized equipment and are less accessible, limiting their practicality in typical clinical settings. Although pelvic floor exams can vary depending on provider technique, they do not have specialized equipment or accessibility barriers, making them easier to implement consistently in standard clinical practice. Integration of exam-findings and patient-reported outcomes in this study provided valuable prospective data on the trajectory of pelvic floor function throughout and after treatment. Missed examinations were primarily related to scheduling logistics, missed clinic visits, or patient preference rather than procedural intolerance. Importantly, no adverse events were attributed to the pelvic examinations, and the majority of patients agreed to repeated assessments over time. These findings suggest that incorporating a brief standardized pelvic floor examination alongside PROM collection is feasible within the clinical workflow of patients undergoing chemoradiotherapy. Although follow-up exam completion declined at later time points, this pattern reflects common challenges in survivorship follow-up rather than limitations of the screening approach itself. Future studies with larger cohorts and structured scheduling protocols may further improve completion rates and confirm the practicality of implementing routine pelvic floor dysfunction screening in gynecologic oncology practice.

Importantly, pelvic floor dysfunction has substantial implications for long-term survivorship in cervical cancer patients. Persistent pelvic floor dysfunction can lead to chronic urinary and bowel symptoms, sexual dysfunction, pelvic pain, and decreased overall quality of life, which are increasingly recognized as critical components of survivorship care (Mohan et al., 2025). By demonstrating that, pelvic floor dysfunction persists in a substantial minority of cervical cancer survivors. Our data suggests an opportunity to improve quality of life in these patients after treatment (Yang et al., 2012 Jun). Routine assessment of pelvic floor function, combined with targeted rehabilitation strategies, may help mitigate long-term disease and enhance functional recovery (Cyr et al., 2020, de Bem Fretta et al., 2026). Integrating pelvic floor dysfunction screening into standard oncology follow-up could therefore support survivorship care, addressing not only disease control but also the physical and psychosocial well-being of cervical cancer survivors. Our results align with prior studies in gynecologic oncology and other populations, which report high prevalence of pelvic floor dysfunction in women with pelvic malignancies, particularly those undergoing pelvic radiation (Cui et al., 2023 Jun, Coughenour et al., 2024 Oct). Existing evidence from gynecologic cancer survivors suggests that pelvic floor physical therapy (PFPT) can reduce dyspareunia, enhance pelvic floor muscle strength, and improve patient-reported quality of life, although data specifically in cervical cancer patients undergoing chemoradiotherapy remain limited (Yang et al., 2012 Jun). Incorporating early PFPT referrals into survivorship care pathways could capitalize on the observed window of functional improvement and reduce long-term morbidity.

Limitations to this study include the small sample size, single-center design, and six-month follow-up, which may limit generalizability. The study was underpowered to perform formal subgroup analyses or detect small effect sizes. Potential biases include selection bias, measurement bias, and missing follow-up assessments. Missing follow-up assessments are unlikely to have meaningfully skewed results with only two, 6-month follow-up PROMs missing. Additionally, despite sequential sampling, we were able to recruit a diverse group of patients eligible for curative intent radiotherapy. Since we did not have the ability to assess participants before cancer diagnosis, we cannot distinguish between pelvic floor dysfunction caused by cancer and pre-existing pelvic floor dysfunction from other causes.

Future research should focus on larger, multi-center cohorts to confirm these findings, evaluate the long-term impact of pelvic floor dysfunction on survivorship outcomes, and determine whether early intervention with pelvic floor rehabilitation during or immediately after treatment can improve patient-reported outcomes, including pain intensity and narcotic use. Additional studies should explore which patient and tumor characteristics such as age, FIGO stage, menopausal status, baseline pelvic floor tone, etc. predict persistence of dysfunction to guide care. Overall, our study demonstrates that pelvic floor dysfunction is prevalent but not immutable in cervical cancer patients undergoing chemoradiotherapy, emphasizing the importance of integrating both objective and subjective assessments of pelvic floor health into comprehensive cancer care.

## CRediT authorship contribution statement

**Sarah N. Badwan:** Writing – review & editing, Writing – original draft, Project administration, Methodology, Investigation, Formal analysis, Data curation. **Tyler R. McKinnish:** . **L.Stewart Massad:** . **Stephanie Markovina:** . **Jessika Contreras:** . **Julie Schwarz:** . **Karry Brim:** . **Brooke E. Sanders:** . **Lindsay M. Kuroki:** . **Andrea R. Hagemann:** . **Carolyn K. McCourt:** . **Premal H. Thaker:** .

## Declaration of competing interest

The authors declare the following financial interests/personal relationships which may be considered as potential competing interests: [Premal H. Thaker reports a relationship with Imunon Inc that includes: consulting or advisory and equity or stocks. Premal H. Thaker reports a relationship with Glaxo Smith Kline that includes: consulting or advisory and funding grants. Premal H. Thaker reports a relationship with AstraZeneca LP that includes: consulting or advisory and funding grants. Premal H. Thaker reports a relationship with Genelux that includes: consulting or advisory. Premal H. Thaker reports a relationship with Verastem Inc that includes: consulting or advisory and travel reimbursement. Premal H. Thaker reports a relationship with Corcept Therapeutics Inc that includes: consulting or advisory. Premal H. Thaker reports a relationship with Merck & Co Inc that includes: consulting or advisory and funding grants. Premal H. Thaker reports a relationship with Incyte Corporation that includes: consulting or advisory and travel reimbursement. Premal H. Thaker reports a relationship with BioNTech US Inc that includes: consulting or advisory. Premal H. Thaker reports a relationship with Caris Life Sciences Inc that includes: consulting or advisory. Premal H. Thaker reports a relationship with Up To Date that includes: consulting or advisory. I am on the editorial board of Gynecologic Oncology. If there are other authors, they declare that they have no known competing financial interests or personal relationships that could have appeared to influence the work reported in this paper].

## Appendix A Supplementary data

Supplementary Data 1 Full set of patient-reported outcome measure (PROM) questionnaires utilized in this study

Supplementary Data 2 Line graph illustrating changes in total pelvic examination scores across study time points, representing summed scores for bilateral obturator internus and levator ani muscle groups.
