# Supplementary figures and images for: Correlation of patient reported outcomes and physical exam screening for pelvic floor dysfunction: An opportunity for improved quality of life during chemoradiotherapy for cervical cancer
Source: Gynecol Oncol Rep. 2026 Apr 19;65:102083. doi: 10.1016/j.gore.2026.102083 (PMC13127208; doi:10.1016/j.gore.2026.102083)

S1. PROMs


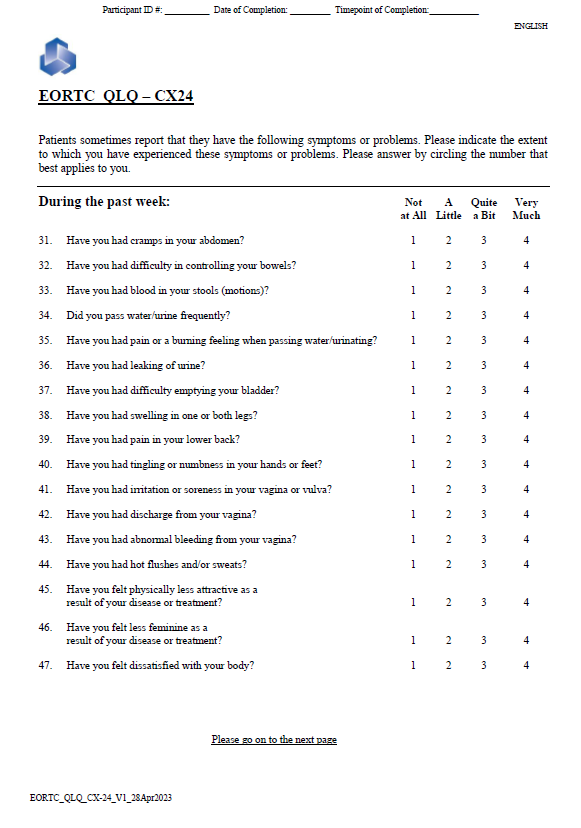


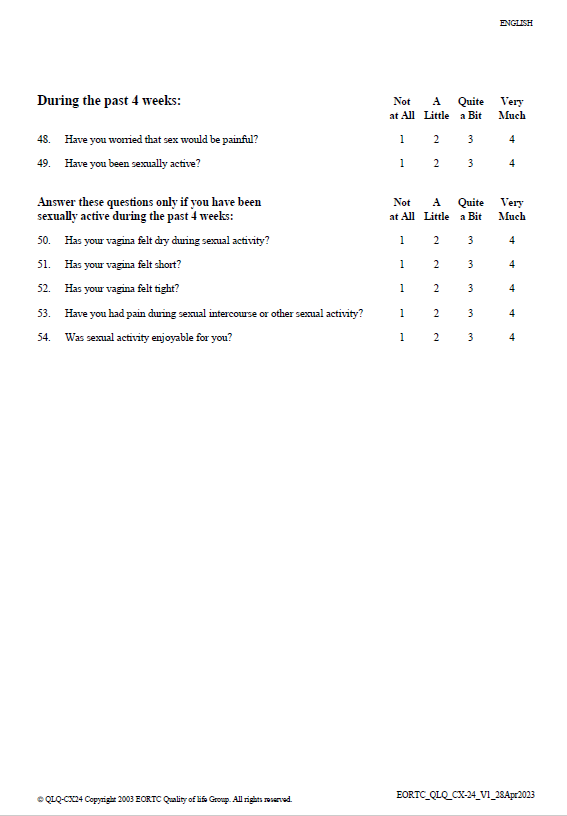


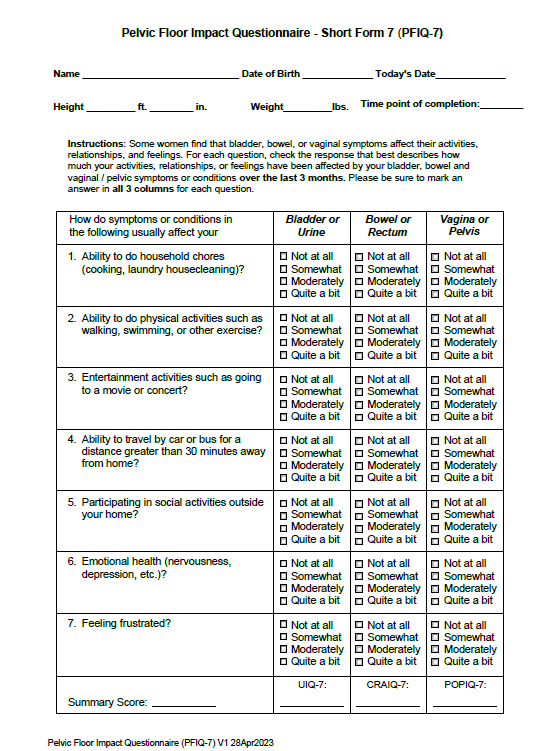


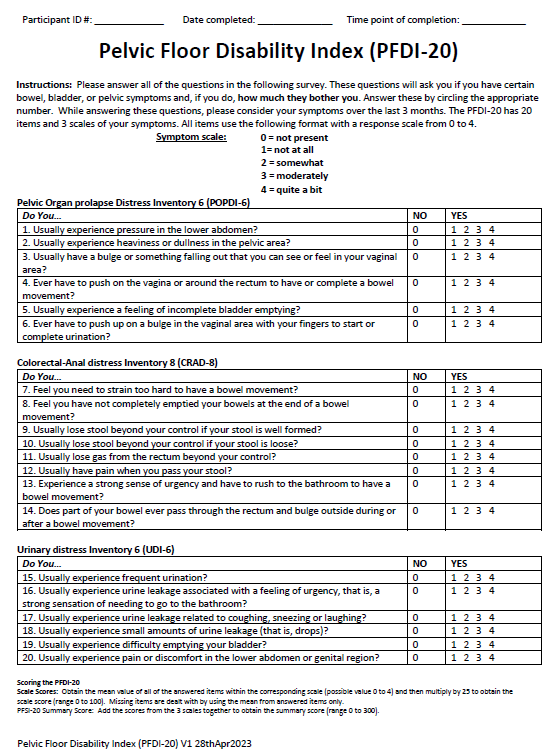

Supplement: Supplementary Data 1 — Full set of patient-reported outcome measure (PROM) questionnaires utilized in this study [file mmc1.docx]

S2. – Exam Scores Over Time


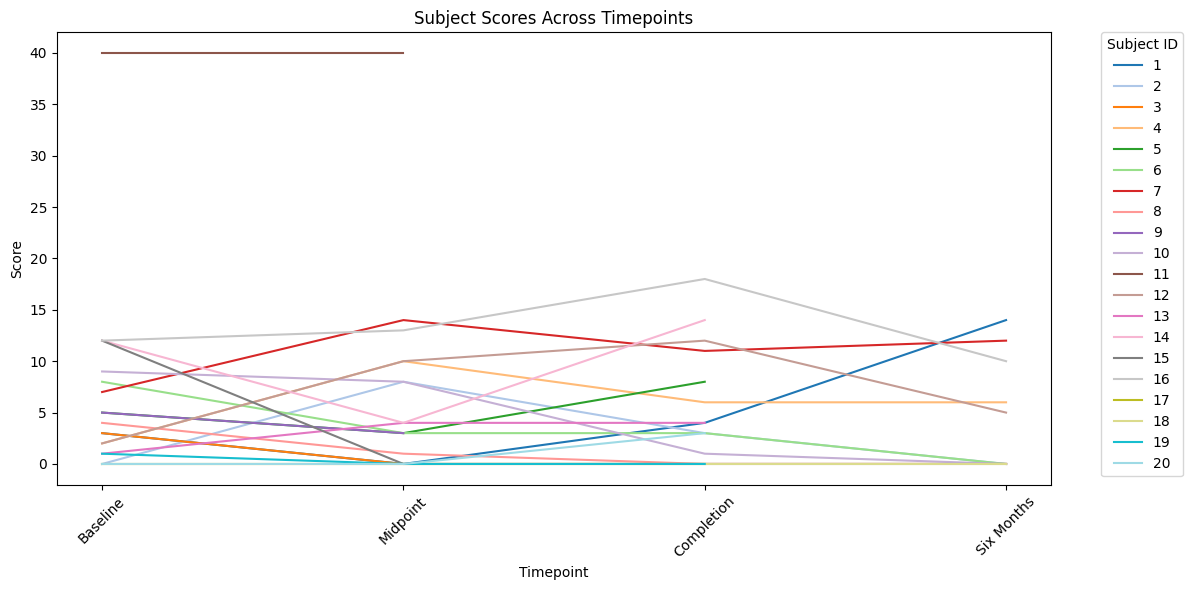

Supplement: Supplementary Data 2 — Line graph illustrating changes in total pelvic examination scores across study time points, representing summed scores for bilateral obturator internus and levator ani muscle groups. [file mmc2.docx]
